# Supplementary material for: Ex vivo engineered human plasma cells exhibit robust protein secretion and long-term engraftment in vivo
Source: Nat Commun. 2022 Oct 16;13:6110. doi: 10.1038/s41467-022-33787-8 (PMC9573882; doi:10.1038/s41467-022-33787-8)
Supplement: Supplementary file 1 — Supplementary Information [file 41467_2022_33787_MOESM1_ESM.pdf]

SUPPLEMENTARY INFORMATION

Supplementary Table 1. Antibodies and kits.

Supplementary Table 2. Parameters used in fitting equation 3.

Supplementary Notes: Model 1, Model 2.

Supplementary Figures. 1 to 10

Supplementary Table 1. Antibodies and kits.

Experiment	Item	Vendor	Catalog	Dilution fold
Flow cytometry	PE-Cy7 anti-human CD19	Biolegend	302216	200X
	PerCP-Cy5.5 anti-human CD38	BD	BDB551400	200X
	PE anti-human CD138	Biolegend	356504	100X
	PacBlue anti-human IgM	Biolegend	314514	300X
	APC anti-human IgM	BD	551062	300X
	APC-Vio 770 IgA Antibody, anti-human	Miltenyi	130-113-473	200X
	AF700 anti-human IgG	BD	561296	200X
	PE-Cy7 Mouse Anti-Human CD45	BD	557748	200X
	Brilliant Violet 421 Anti-Human CD45	BD	563879	200X
	Brilliant Violet 605 anti-mouse CD45	Biolegend	103140	200X
	Brilliant Violet 510 anti-human CD19	Biolegend	302242	200X
	FITC anti-BrdU	BD	347583	50X
CITESEQ	TotalSeq-B0251 anti-human Hashtag 1 Antibody	Biolegend	394631	1000X
	TotalSeq-B0251 anti-human Hashtag 2 Antibody	Biolegend	394633	1000X
	TotalSeq-B0251 anti-human Hashtag 3 Antibody	Biolegend	394635	1000X
	TotalSeq-B0251 anti-human Hashtag 4 Antibody	Biolegend	394637	1000X
	TotalSeq-B0136 anti-human IgM Antibody	Biolegend	314549	200X
	TotalSeq-B0389 anti-human CD38 Antibody	Biolegend	303547	800X

	TotalSeq-B0055 anti-human CD138 (Syndecan-1) Antibody	Biolegend	356543	100X
ELISPOT	Goat Anti-Human IgG-HRP	Southern Biotech	2040-05	1000X
	Anti-human IgG	ThermoFisher	A18813	250X
	AEC Substrate Kit, Peroxidase (HRP), (3-amino-9-ethylcarbazole)	Vector Laboratories	SK4200	
ELISA	Invitrogen IgG (Total) Human Uncoated ELISA Kit	Invitrogen	50-112-8849	
	Invitrogen IgM (Total) Human Uncoated ELISA Kit	Invitrogen	50-112-8719	
	ELISA MAX Deluxe Set Human IL-6	Biolegend	430504	
	Human BAFF/BLyS/TNFSF13B DuoSet ELISA	R&D	DY124-05	

Supplementary Table 2. Parameters used in fitting equation 3.

	Antibody secretion rate / volume of displacement	Antibody degradation rate	IgG	PCs decay rate	PC Initial number
	$\gamma(ng \cdot cell^{-1} \cdot day^{-1})$ / 2 ml	$\alpha\ (day^{-1})$	$G\ (ng/ml)$	$\beta\ (day^{-1})$	$N_0(cell)$
Mock	0.1 (adjusted: 0.75)	0.495	Experimental data	fitting	fitting
hIL6	0.1	0.495	Experimental data	fitting	fitting

SUPPLEMENTARY NOTE

Model 1:

The number of ASCs and hIgG immediately following cell transfer is hard to model due to the following reasons: (1) Post-transfer, much of the inoculum accumulates in the lung and liver. A large percentage of transferred B cells are subsequently cleared by the host, potentially leaving only a small percentage of surviving cells. (2) We expect a portion of the cell inoculum to differentiate following *in vivo* transfer. (3) *In vitro* experiments indicate that the hIgG secretion rate, γ, is approximately steady 21 days following *ex vivo* differentiation (Fig. 6h). Therefore, for the purposes of this model, we fixed γ as a parameter. Finally, based on BRDU labeling of transferred cells, terminally differentiated PCs do not divide at a measurable rate; therefore in this model, we solely consider their death rate (decay).

Let us denote the number of PC as N and the amount of antibody hIgG as G. Their dynamic can be modeled by the following equations.

$$\frac{dN}{dt} = -\beta N \quad (1)$$

$$\frac{dG}{dt} = \gamma N - \lambda G = \gamma N_0 e^{-\beta t} - \lambda G \quad (2)$$

where β represents the PC decay rate, γ represents the hIgG secretion rate, and λ represents the clearance rate of hIgG. The solution of eq.2, the amount of antibody at a give time (t) is

$$G(t) = \frac{\gamma N_0}{\lambda - \beta}\left(e^{-\beta t} - e^{-\lambda t}\right) \quad (3)$$

The parameters γ, λ are described previously [1–4]. We used Eq. 3to model the PC behavior and fit the initial number of PCs that survive transit through the lung/liver, and/or differentiate and survive *in vivo* beyond 21 days (denoted as N_0) and the decay rate (β) of these cells. In this model, we explicitly ignore short-lived PCs that produce antibodies initially following transfer, but are not stably retained.

Model 2:

To uncover how the hIL6 affects the β of PCs in the short-term, we assumed that β is determined by two factors: proliferation rate and death rate, denoted as α and δ (Fig. 6a), respectively. In models of short-term *in vitro* studies, we found that β as a parameter is only able to fit the first 6 days of observation (Fig. 6c, dash line). That is, the decay rate (β) of PCs immediately following *ex vivo* differentiation during the first 21 days of our observation changes with time. Notice that $\beta(t) = -dlnN/dt$, where the latter (change in PC number over time) can be estimated from the counting data (Supplementary Fig. 8). We observed that $dlnN/dt$ has a linear-like decay. Therefore, we use $\beta(t) = \beta_0 + \beta_1{}'t$ to characterize the changes in decay rate over time and fit β_0 and $\beta_1{}'$. The ODE solution of $N(t)$ is,

$$N(t)/N_0 = e^{-\ (\beta_0 t + \beta_1 t^2)} \quad (4)$$

The resulting dynamics of fitted β_0 and β_1 matched the data (Fig. 6c, solid line), and we presented all the fitted dynamics of all samples with hIL6 and without hIL6 (Fig. 6b). β_0 is the initial value of β. It can be interpreted as the acute effect of hIL6. β_1 is the slope of $\beta(t)$, characterizing the changes in decay rate over time. We observed that the fitted β_1 (Fig. 6d) are negative, demonstrating that PC decay rate decreased over time (Supplementary Fig. 8). Here, negative

$d\beta/dt$ can be explained by two possible factors. (1) The PC cells are initially composed of subpopulations with different decay rates $\{\beta^{(1)}, \beta^{(2)}, \beta^{(3)}, \ldots, \beta^{(m)}\}$, and based on Fisher's fundamental theorem of nature selection (FTNS), the decay rate becomes smaller as cells with larger proliferation rate or smaller death rate will dominate the population overtime [5]. (2) A subset of PCs can mature in culture and differentiate into long-lived PCs over time $\{\beta^{(1)}(t), \beta^{(2)}(t), \beta^{(3)}(t), \ldots, \beta^{(m)}(t)\}$, so intrinsically the decay rate is changing. We found that the initial value of decay rate, β_0, is significantly affected by hIL6 (p-value = 0.003) (Fig. 6d). The distributions of β with or without hIL6 for the first 6 days are prominently separated (Supplementary Fig. 8).

β_0's predominant difference aligns with the drastic change of $E[firefly\ luciferase_{IL6+}]/E[firefly\ luciferase_{IL6-}]$ for the first 3 days *in vivo* (Fig. 5b). We further show that the slope of β, characterized by β_1, is also different (Fig. 6d, p-value = 0.03). The difference in β_1 shows the β eventually converge to a similar level at day 21 even with significant difference at the beginning, which matches the closeness of β from the long-term model (Fig. 4i). Therefore, we see the converged decay rate from the two systems (hIL6 versus vehicle). The linear model of β can characterize the decay of β in 21 days as well. However, at the later stage of the decay, we observed that hIL6 does not affect β at later time points, although it is possible that other human cytokines or molecules would decrease cellular decay following establishment of populations of long-lived PCs.

SUPPLEMENTARY FIGURES

Supplementary Figure 1. CITE-seq RNAseq

(a) Single cell trajectory graph heatmap of total counts gene expression and surface protein tags (left panel). Histogram (middle) and heatmap (right) of hashtag oligos (HTO) from different samples after demultiplexing by Hashsolo. **(b)** Expression heatmap of top genes differential from RNA Leiden clusters (in Fig. 1g). **(c)** UMAP graph heatmap showing expression of immunoglobulin genes. **(d)** Gene set analysis (GSEA): Biological process functional annotation and cellular component annotation (right) of enriched genes from CD38hi (left). GSEA online analysis: http://www.webgestalt.org/ (**e**) Graph showing the scanpy scores (Z-score of the gene level p-value) of the top 50 highly enriched genes in CD138^{hi} PCs.

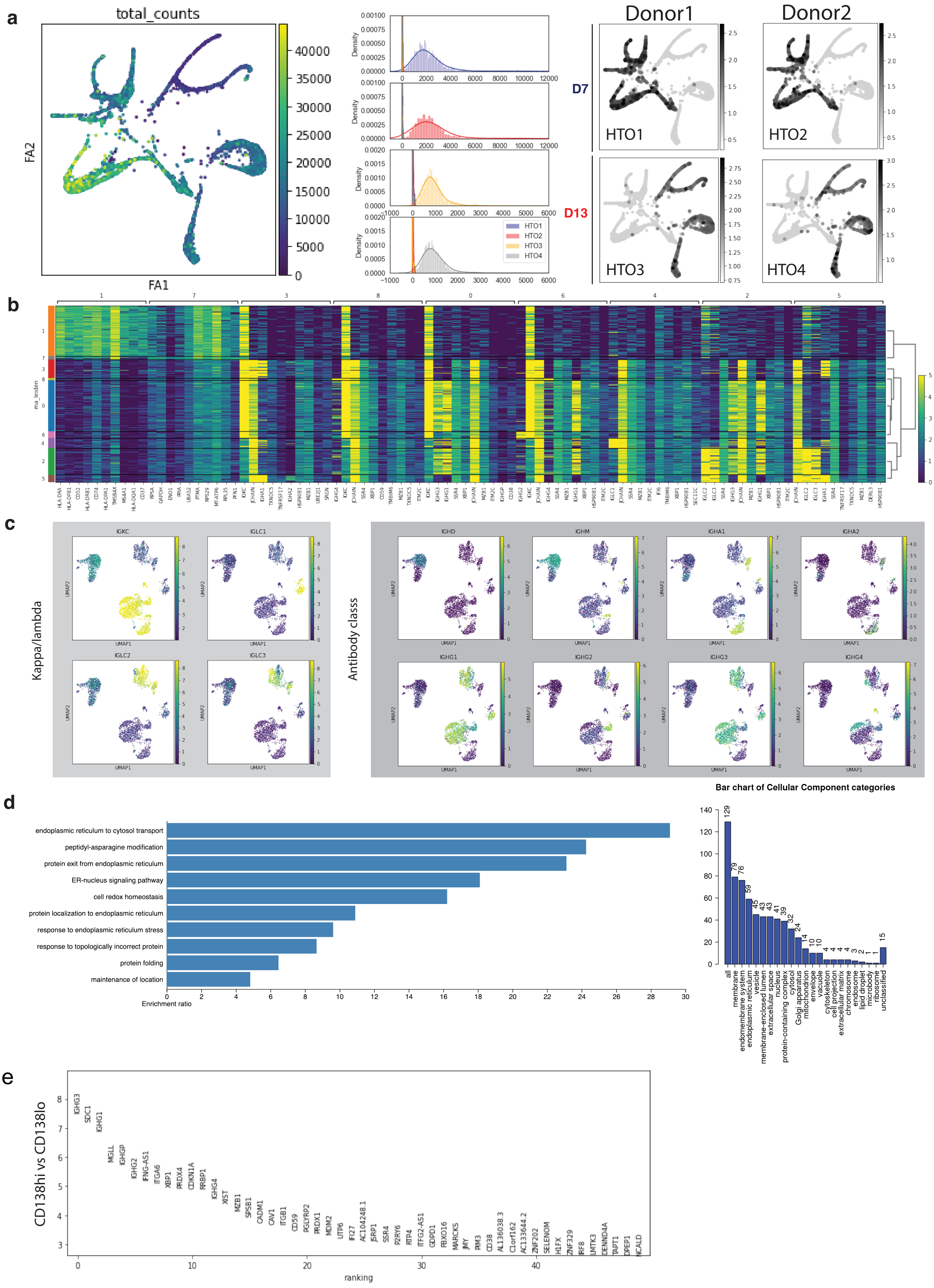
a
total_counts
FA2
FA1
Density
HTO1
HTO2
HTO3
HTO4
Donor1
Donor2
D7
D13
b
ma_leiden
c
Kappa/lambda
IGKC
IGLC1
IGLC2
IGLC3
Antibody class
IGHD
IGHM
IGHA1
IGHA2
IGHG1
IGHG2
IGHG3
IGHG4
UMAP1
UMAP2
d
endoplasmic reticulum to cytosol transport
peptidyl-asparagine modification
protein exit from endoplasmic reticulum
ER-nucleus signaling pathway
cell redox homeostasis
protein localization to endoplasmic reticulum
response to endoplasmic reticulum stress
response to topologically incorrect protein
protein folding
maintenance of location
Enrichment ratio
Bar chart of Cellular Component categories
all
membrane
endomembrane system
endoplasmic reticulum
vesicle
membrane-enclosed lumen
extracellular space
nucleus
protein-containing complex
cytosol
Golgi apparatus
mitochondrion
envelope
vacuole
cytoskeleton
cell projection
extracellular matrix
chromosome
endosome
lipid droplet
microbody
ribosome
unclassified
e
CD138hi vs CD138lo
ranking
IGHG3
SDC1
IGHG1
MGLL
IGHGP
IGHG2
IFNG-AS1
ITGA6
XBP1
PRDX4
CDKN1A
RRBP1
IGHG4
XIST
MZB1
SPSB1
CADM1
CAV1
ITGB1
CD59
PGLYRP2
PRDX1
MDM2
UTP6
IFI27
AC104248.1
JSRP1
SSR4
P2RY6
RTP4
ITFG2-AS1
GDPD1
FBXO16
MARCKS
JMY
PIM3
CD38
AL136038.3
C1orf162
AC133644.2
ZNF202
SELENOM
H1FX
ZNF329
IRF8
LMTK3
DENND4A
TAPT1
DPEP1
NCALD

Supplementary Figure 2. Mitochondria staining and TEM

(a) Gating strategy for B cell subsets. **(b)** Flow cytometry showing expression of MitoTracker Green FM (mitochondrial volume) and MitoTracker Red CMXRos (mitochondrial stress) from different B cell subsets from 5 different donors. Data normalized by scaling input vectors individually to unit norm. **(c)** Representative transmission electron microscopy of day 2 and day 13 B cells from three-stage culture *ex vivo*. Data selected from 43 images in day 2 cells and 60 images in day 13 cells.

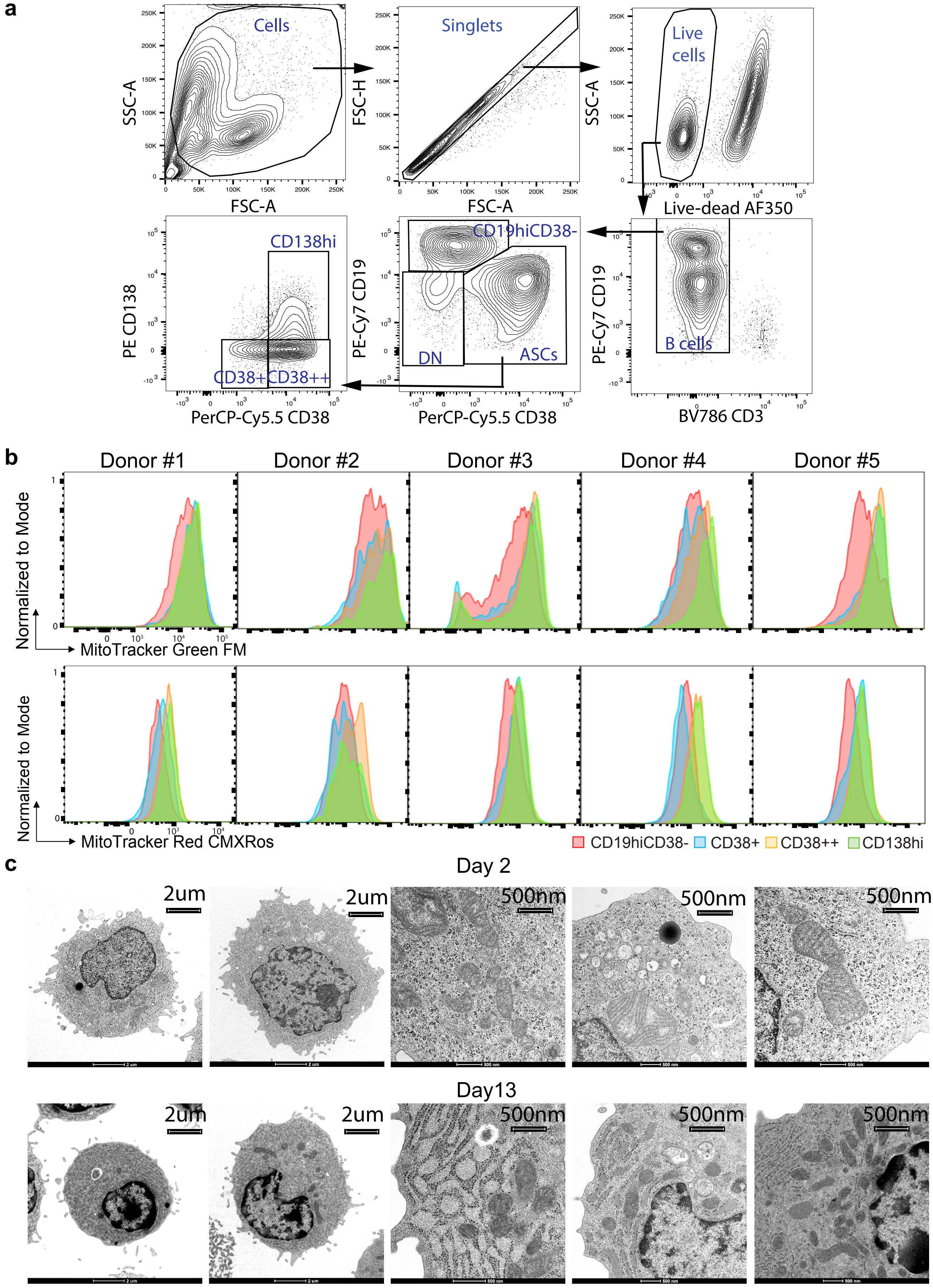
a
Cells
Singlets
Live cells
SSC-A
FSC-A
FSC-H
Live-dead AF350
CD138hi
CD19hiCD38-
PE CD138
PE-Cy7 CD19
DN
ASCs
B cells
CD38+
CD38++
PerCP-Cy5.5 CD38
BV786 CD3
b
Donor #1
Donor #2
Donor #3
Donor #4
Donor #5
Normalized to Mode
MitoTracker Green FM
MitoTracker Red CMXRos
CD19hiCD38-
CD38+
CD38++
CD138hi
c
Day 2
Day13
2um
500nm

Supplementary Figure 3. *Ex vivo* differentiated PCs home to BM and secrete hIgG for up to 1 year.

(a) Firefly luciferase gene was introduced by the HDR strategy into B cell *CCR5* locus at day 2 of three-stage culture, and *in vivo* engraftment tracking engineered B cells from Day 0 to day 51 using firefly luciferase imaging by IVIS (The imaging data is representative from a single donor; donor 2). **(b)** hIgG was quantified in serum from Day 0 to day 51 by ELISA (donor 2, n=5 animals). The line indicates the mean at each time point, and the shadow shows the 95% confidence intervals. Source data are provided as a Source Data file. **(c)** hIgG was quantified in serum by ELISA for up to 1-year post-transfer (n = 4 animals). The line indicates the mean at each time point, and the shadow shows the 95% confidence intervals. Source data are provided as a Source Data file. **(d)** Dotplot visualization of day 13 B cells (n= 2897 cells): subsets are listed on y-axis and genes are listed along the x-axis. Dot size represents percentage of cells in a group expressing each gene; dot color indicates mean expression level in a group. **(e)** A heatmap showing expression of representative genes from the RNA sequencing analysis from the CD138+ and CD38+CD138- fractions from 4 subjects (columns). **(f)** Gene counts from the indicated genes from the CD138+ and CD38+CD138- from 4 subjects at day 13 or day 19 in culture are presented (n = 4 subjects).

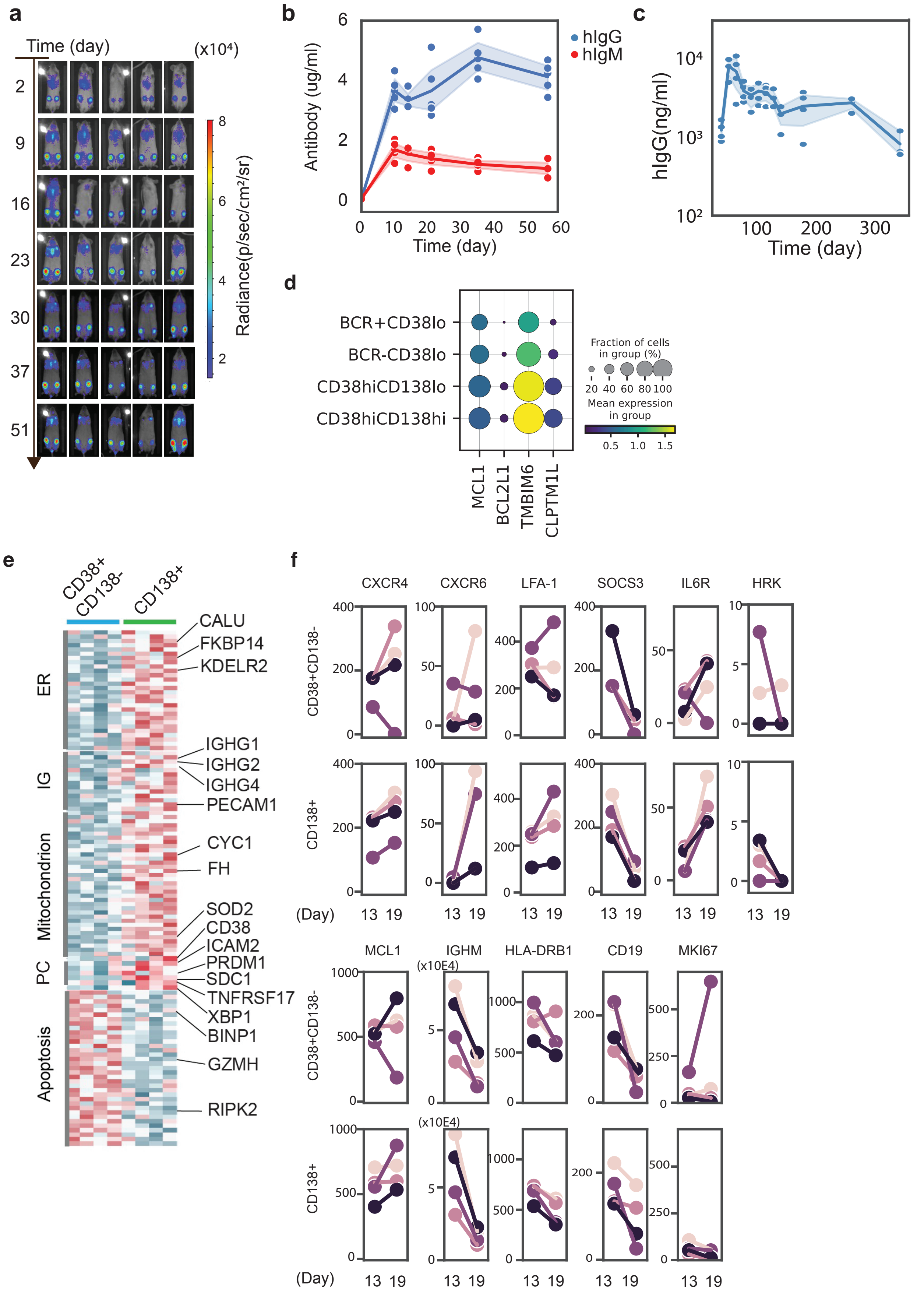

a
Time (day)
(x10⁴)
2
9
16
23
30
37
51
Radiance(p/sec/cm²/sr)
b
Antibody (ug/ml)
Time (day)
hIgG
hIgM
c
hIgG(ng/ml)
Time (day)
d
BCR+CD38lo
BCR-CD38lo
CD38hiCD138lo
CD38hiCD138hi
MCL1
BCL2L1
TMBIM6
CLPTM1L
Fraction of cells in group (%)
Mean expression in group
e
CD38+ CD138-
CD138+
ER
IG
Mitochondrion
PC
Apoptosis
CALU
FKBP14
KDELR2
IGHG1
IGHG2
IGHG4
PECAM1
CYC1
FH
SOD2
CD38
ICAM2
PRDM1
SDC1
TNFRSF17
XBP1
BINP1
GZMH
RIPK2
f
CXCR4
CXCR6
LFA-1
SOCS3
IL6R
HRK
CD38+CD138-
CD138+
(Day)
MCL1
IGHM
HLA-DRB1
CD19
MKI67
(x10E4)

Supplementary Figure 4. PC protein secretion in hIL6-engineered mice and hBAFF-edited cells

(a) hIL6 levels in hIL6-NSG mice serum before cell transplantation 11 days. Source data are provided as a Source Data file. **(b)** Ratio of the mean of hIgG in hIL6-NSG (n=5 animals) to that of NSG mice (n=4 animals). The horizontal line represents the mean of the fold change across all timepoints. **(c)** Pearson correlation between hIL6 and human antibodies in mice serum quantified by ELISA at last two time points (t = 266 day and 357 day), each dot represents one animal (n=8 animals). **(d, e)** ELISA was used to quantify hIL6 in serum and B cell production of hIgM in NSG or NSG-hIL6 animals with unedited B cells and hBAFF-edited B cells. Data collected from unedited PC in control mice (n=4 animals), unedited PC in hIL6 mice (n=4 animals), hBAFF-edited PC in control mice (n=5 animals) and hBAFF-edited PC in hIL6 mice (n=5 animals). Source data are provided as a Source Data file.

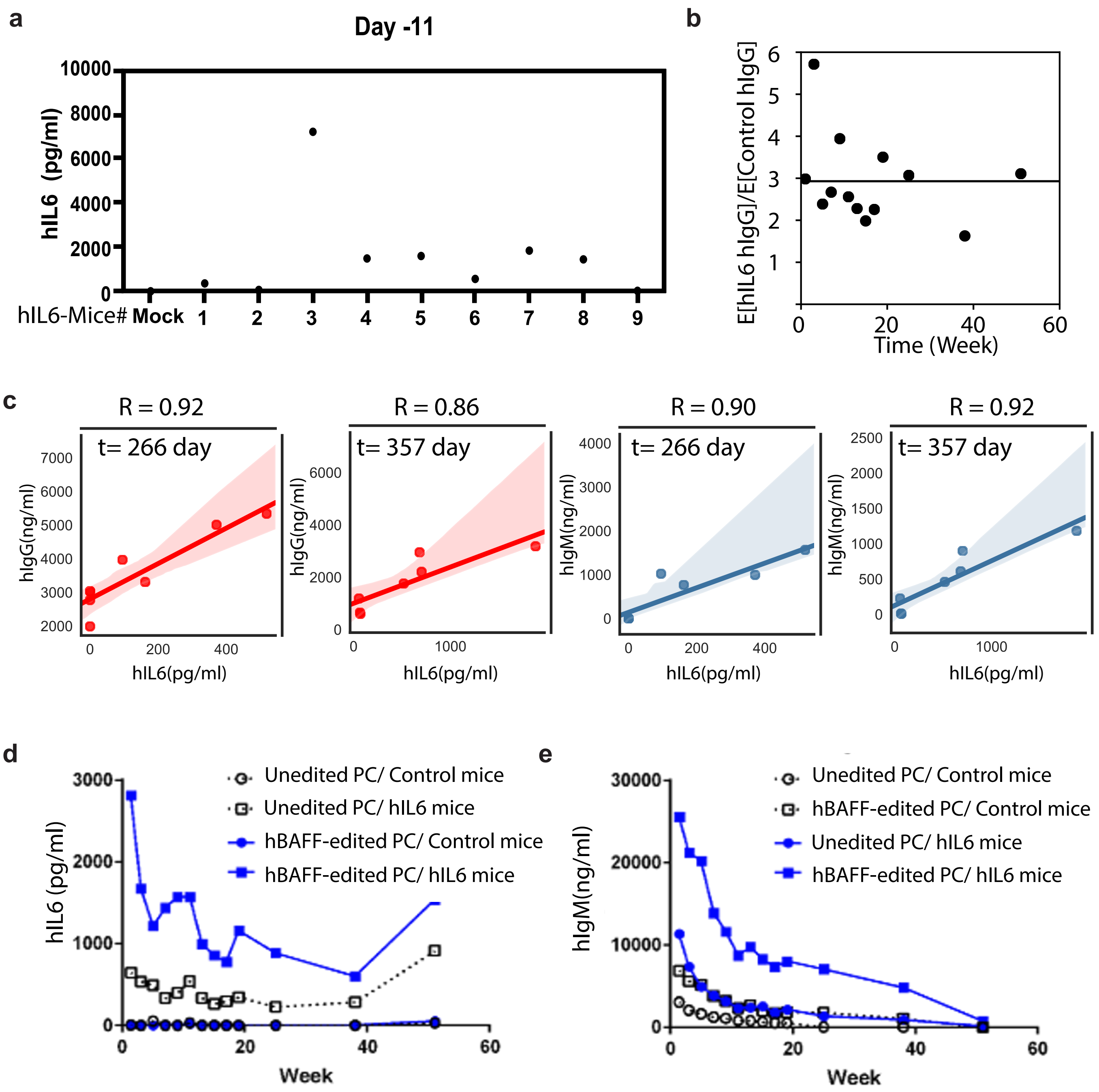

a
Day -11
hIL6 (pg/ml)
10000
8000
6000
4000
2000
0
hIL6-Mice# Mock 1 2 3 4 5 6 7 8 9
b
E[hIL6 hIgG]/E[Control hIgG]
Time (Week)
c
R = 0.92
t= 266 day
hIgG(ng/ml)
hIL6(pg/ml)
R = 0.86
t= 357 day
hIgG(ng/ml)
hIL6(pg/ml)
R = 0.90
t= 266 day
hIgM(ng/ml)
hIL6(pg/ml)
R = 0.92
t= 357 day
hIgM(ng/ml)
hIL6(pg/ml)
d
hIL6 (pg/ml)
Unedited PC/ Control mice
Unedited PC/ hIL6 mice
hBAFF-edited PC/ Control mice
hBAFF-edited PC/ hIL6 mice
Week
e
hIgM(ng/ml)
Unedited PC/ Control mice
hBAFF-edited PC/ Control mice
Unedited PC/ hIL6 mice
hBAFF-edited PC/ hIL6 mice
Week

Supplementary Figure 5. hBAFF increases the number of IgG ASCs.

(a) Day 13 phenotype of hBAFF-edited B cells. Cells were stained with the indicated antibody markers. Cells were gated into GFP+ (hBAFF edited cells) and GFP- (unedited cells) and the marker expression was visualized using a tSNE plot. The color codes were indicated by the labels. **(b)** Stacked bar chart representing flow cytometry quantification of hBAFF-edited and unedited cells using the indicated antibodies.

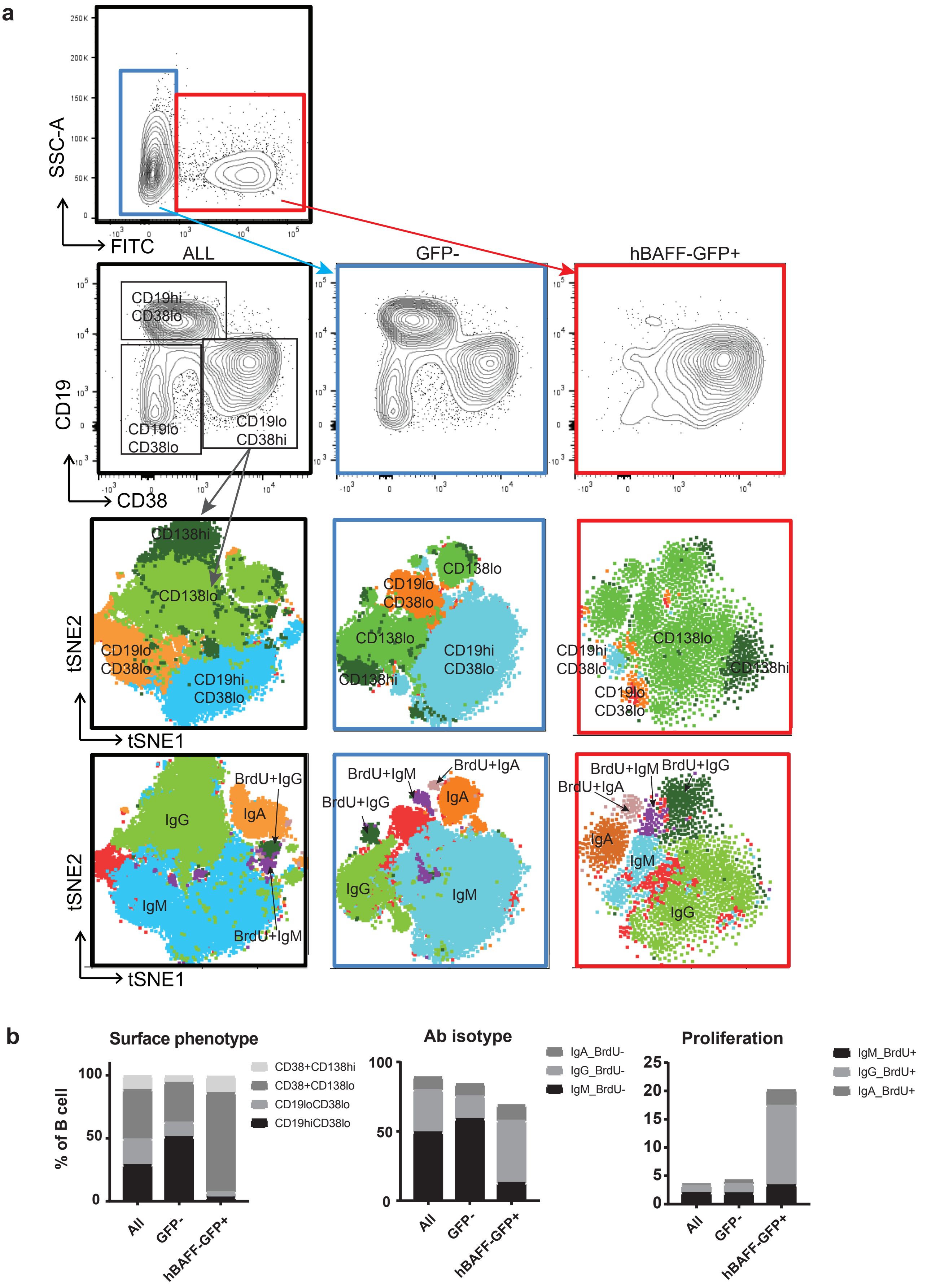

a
SSC-A
FITC
ALL
GFP-
hBAFF-GFP+
CD19hi CD38lo
CD19lo CD38lo
CD19lo CD38hi
CD19
CD38
CD138hi
CD138lo
tSNE2
tSNE1
IgG
IgA
IgM
BrdU+IgG
BrdU+IgM
BrdU+IgA
b
Surface phenotype
% of B cell
CD38+CD138hi
CD38+CD138lo
CD19loCD38lo
CD19hiCD38lo
Ab isotype
IgA_BrdU-
IgG_BrdU-
IgM_BrdU-
Proliferation
IgM_BrdU+
IgG_BrdU+
IgA_BrdU+
All
GFP-
hBAFF-GFP+

Supplementary Figure 6. Model for longevity of engrafted PCs predicts dynamics in mice.

(a) Schematic of model fitting for hIgG dynamics in long and short-lived ASCs (model 1, Supplementary note). **(b)** Estimated engrafted cell number and decay rate by model 1 in NSG mice engrafted with unedited (n=4 animals) and hBAFF-edited cells (n=5 animals). **(c)** Long-lived PC cell numbers at the last time point as predicted by the model, equation 1 Supplementary note. **(b, c)** Box plot represents a central line denoting median value (50th percentile), while the box contains the 25th to 75th percentiles of the dataset with black whiskers marking the maximum (95th percentile) and minimum (5th percentile). **(d)** Flow cytometry showing the distribution of the indicated surface markers in cells collected from the BM in transfer experiments using unedited and hBAFF-edited cells collected in NSG or hIL6 NSG mice. **(e-f)** PCs engineered to express firefly luciferase were transferred into NSG (n=5 animals) and hIL6-NSG (n=3 animals) mice. The mice were imaged **(e)**, luciferase was quantified **(f)** and IgG levels were quantified **(g)** The shadows represent 95% confidence intervals and lines represent mean. Source data are provided as a Source Data file.

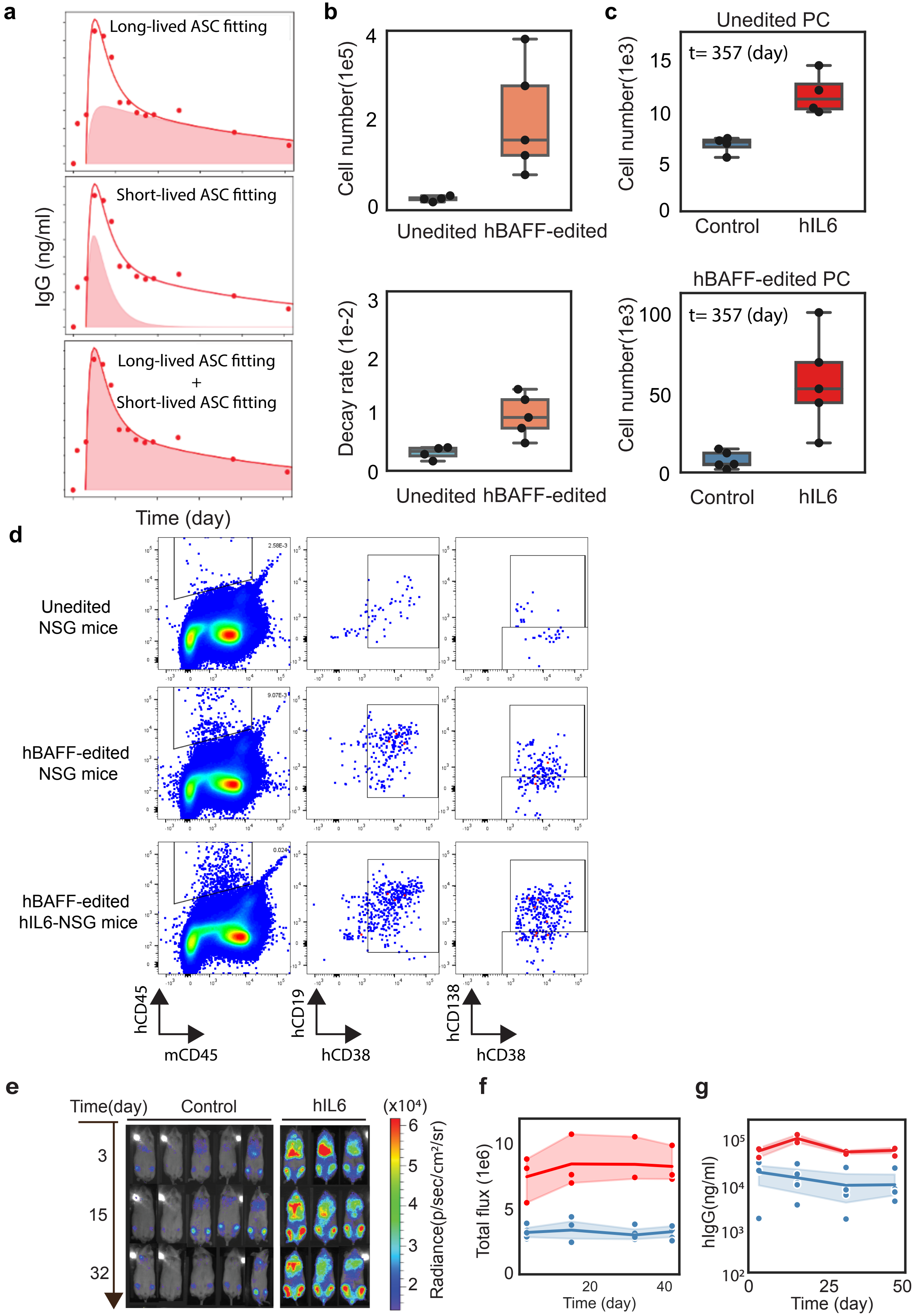
a
Long-lived ASC fitting
Short-lived ASC fitting
Long-lived ASC fitting
+
Short-lived ASC fitting
IgG (ng/ml)
Time (day)
b
Cell number(1e5)
Unedited hBAFF-edited
Decay rate (1e-2)
Unedited hBAFF-edited
c
Unedited PC
t= 357 (day)
Cell number(1e3)
Control hIL6
hBAFF-edited PC
t= 357 (day)
Cell number(1e3)
Control hIL6
d
Unedited NSG mice
hBAFF-edited NSG mice
hBAFF-edited hIL6-NSG mice
hCD45
mCD45
hCD19
hCD38
hCD138
hCD38
e
Time(day)
Control
hIL6
3
15
32
(x10⁴)
Radiance(p/sec/cm²/sr)
f
Total flux (1e6)
Time (day)
g
hIgG(ng/ml)
Time (day)

Supplementary Figure 7. Human B cell engraftment is higher in a humanized IL6 mouse model.

(a) Images from an *in vivo* engraftment experiment tracking engineered B cells from Day 0 to day 28 in hIL6 B-NDG and B-NDG control mice using firefly luciferase imaging by IVIS. **(b)** Representative flow cytometry showing surface expression of the indicated markers on B cell subsets in the BM of hIL6 B-NDG and B-NDG control mice. **(c)** Quantitation of flow cytometry in figure b across hIL6 B-NDG mice (n=8 animals) and control B-NDG mice (n=7 animals). Box plot represents a central line denoting median interquartile range median value (50th percentile), while the box contains the 25th to 75th percentiles of the dataset with black whiskers marking the maximum (95th percentile) and minimum (5th percentile).

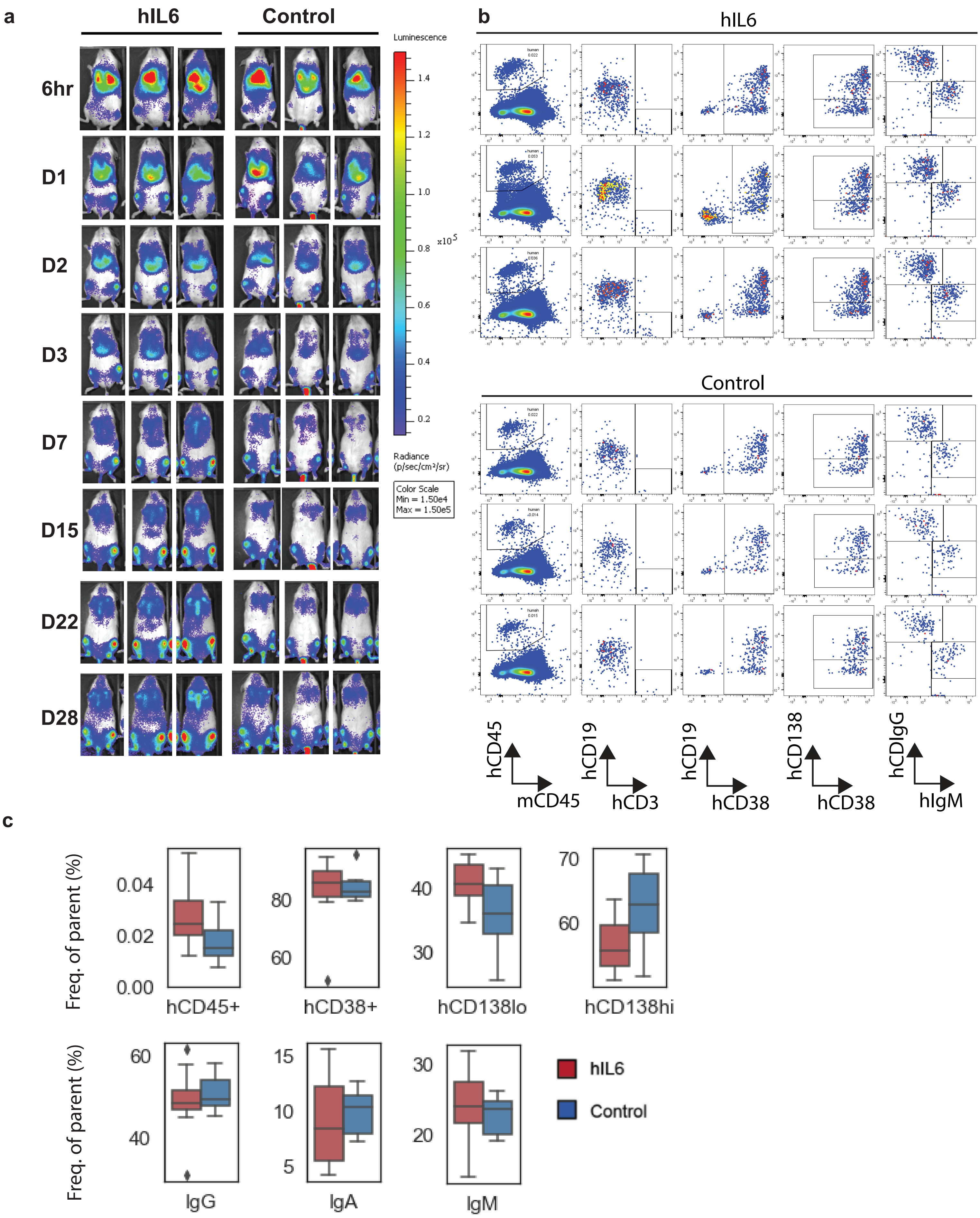
a
hIL6
Control
6hr
D1
D2
D3
D7
D15
D22
D28
Luminescence
1.4
1.2
1.0
0.8
x10^5
0.6
0.4
0.2
Radiance
(p/sec/cm²/sr)
Color Scale
Min = 1.50e4
Max = 1.50e5
b
hIL6
Control
hCD45
mCD45
hCD19
hCD3
hCD19
hCD38
hCD138
hCD38
hCDIgG
hIgM
c
Freq. of parent (%)
0.04
0.02
0.00
hCD45+
80
60
hCD38+
40
30
hCD138lo
70
60
hCD138hi
Freq. of parent (%)
60
40
IgG
15
10
5
IgA
30
20
IgM
hIL6
Control

Supplementary Figure 8. hIL6 decreases the decay rate dynamics of CD138+ cells *ex vivo*

Decay rate of *ex vivo* day 13 CD138+ cells cultured with or without hIL6 for 21 days. Individual decay rates at the indicated time points are represented by dots, and the solid line represents the average decay rate as a function of time. β is fitted by eq.4 in model 2, based on CD138+ cell number dynamics. The line (dim) indicates the mean at each time point, and the shadow shows the 95% confidence intervals.

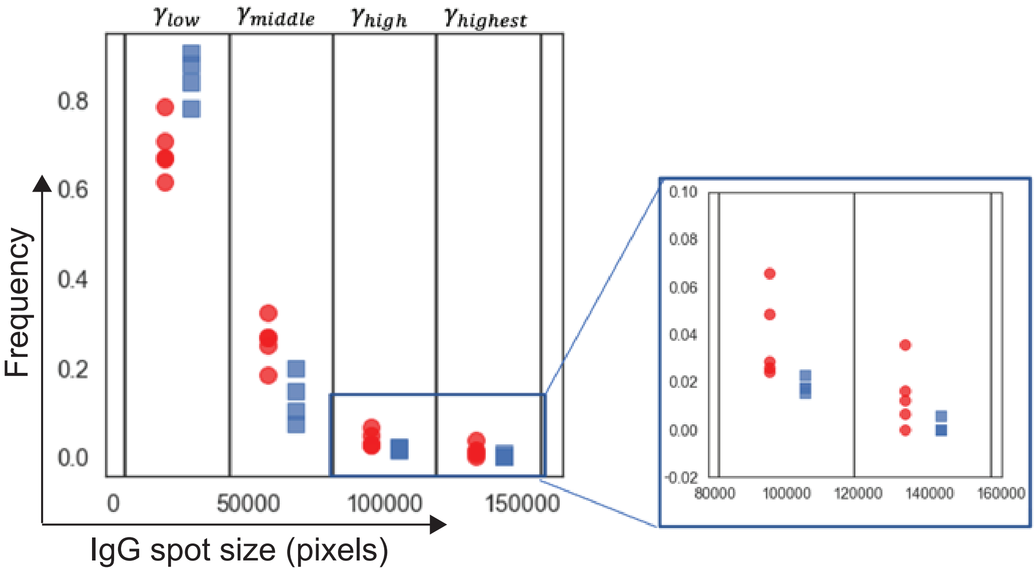

γ_{low} γ_{middle} γ_{high} $\gamma_{highest}$
Frequency
0.0 0.2 0.4 0.6 0.8
0 50000 100000 150000
IgG spot size (pixels)
0.10 0.08 0.06 0.04 0.02 0.00 -0.02
80000 100000 120000 140000 160000

Supplementary Figure 9. hIL6 increases hIgG antibody secretion rate.

Histogram and kernel density estimate (solid line) distributions of ELISPOT size from *ex vivo* day 13 CD138+ cells cultured with or without hIL6 for 21 days. Data was normalized by scaling input vectors individually to unit norm.

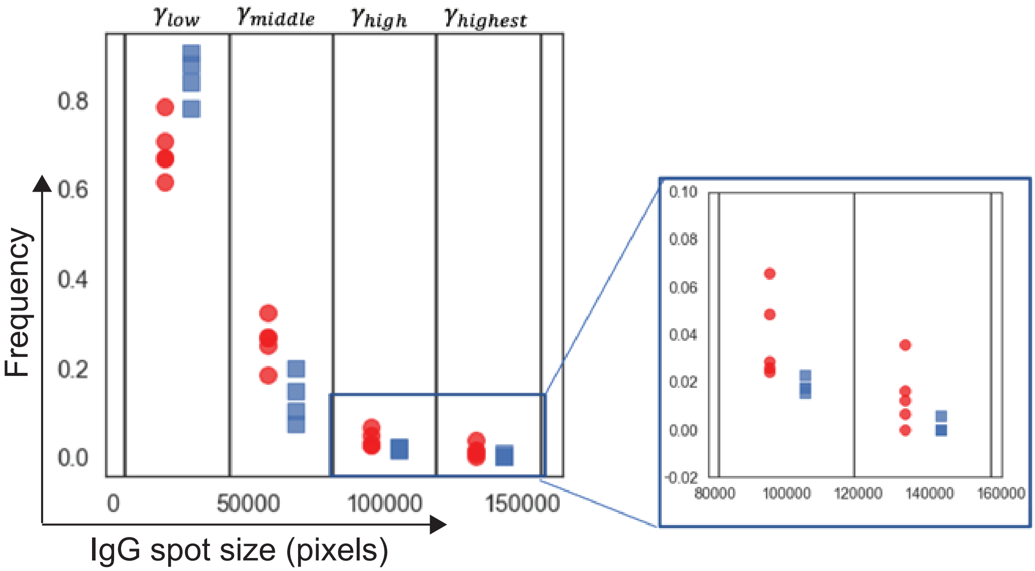

γ_{low}
γ_{middle}
γ_{high}
$\gamma_{highest}$
Frequency
0.0
0.2
0.4
0.6
0.8
0
50000
100000
150000
IgG spot size (pixels)
0.10
0.08
0.06
0.04
0.02
0.00
-0.02
80000
100000
120000
140000
160000

Supplementary Figure 10. Remodeling of long-term *in vivo* experiments

(a, b) Refitted curves of hIgG dynamics in unedited **(a)** and hBAFF-edited **(b)** cells in NSG and hIL6-NSG mice, using adjusted antibody secretion rates (Supplementary Table 2). **(c)** Decay rate and cell engraftment numbers based on re-fitted curves. For each animal, we used the re-fitted curves to calculate decay rate and engrafted PC numbers (Mock, n=4 animals; hBAFF-edited, n=5 animals). Box plot represents a central line denoting median value (50th percentile), while the box contains the 25th to 75th percentiles of the dataset with black whiskers marking the maximum (95th percentile) and minimum (5th percentile). P-values were calculated using an unpaired two-tailed Welch's t-test.

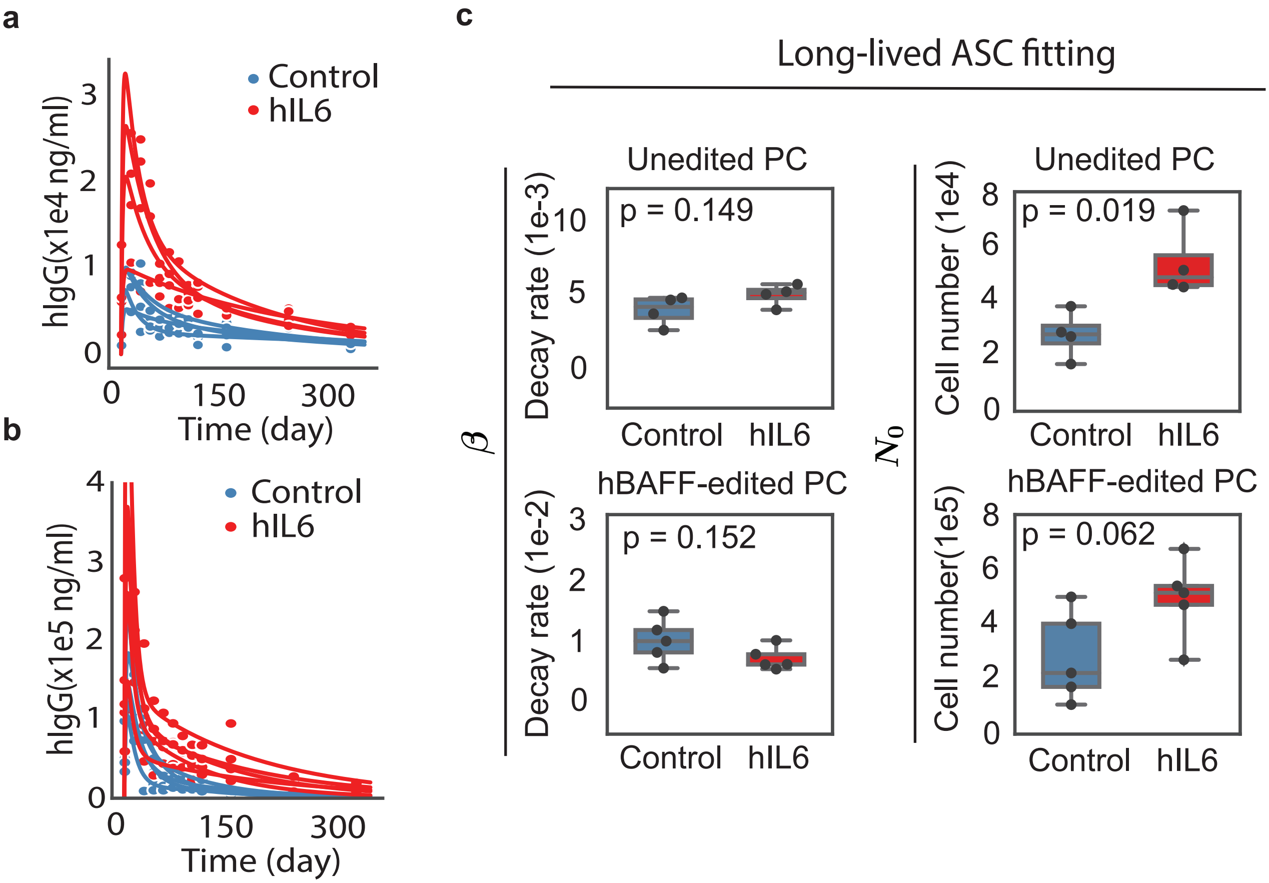
a
Control
hIL6
hIgG(x1e4 ng/ml)
Time (day)
b
Control
hIL6
hIgG(x1e5 ng/ml)
Time (day)
c
Long-lived ASC fitting
β
Unedited PC
Decay rate (1e-3)
p = 0.149
Control
hIL6
hBAFF-edited PC
Decay rate (1e-2)
p = 0.152
Control
hIL6
N0
Unedited PC
Cell number (1e4)
p = 0.019
Control
hIL6
hBAFF-edited PC
Cell number(1e5)
p = 0.062
Control
hIL6

SUPPLEMENTARY REFERENCES

1. Bromage, E., Stephens, R. & Hassoun, L. The third dimension of ELISPOTs: quantifying antibody secretion from individual plasma cells. *J. Immunol. Methods* **346**, 75–79 (2009).
2. Li, F. *et al.* Mouse Strains Influence Clearance and Efficacy of Antibody and Antibody–Drug Conjugate Via Fc–FcγR Interaction. *Mol. Cancer Ther.* **18**, 780–787 (2019).
3. Ghetie, V., Ward, E. S. & Vitetta, E. S. Pharmacokinetics of Antibodies and Immunotoxins in Mice and Humans. in *Handbook of Anticancer Pharmacokinetics and Pharmacodynamics* (eds. Figg, W. D. & McLeod, H. L.) 475–498 (Humana Press, 2004).
4. Salmon, S. E. & Smith, B. A. Immunoglobulin synthesis and total body tumor cell number in IgG multiple myeloma. *J. Clin. Invest.* **49**, 1114–1121 (1970).
5. Fisher, R. A. *The genetical theory of natural selection*. (Рипол Классик).
